# Tenth Scientific Biennial Meeting of the Australasian Virology Society—AVS10 2019

**DOI:** 10.3390/v12060621

**Published:** 2020-06-06

**Authors:** Karla J. Helbig, Rowena A. Bull, Rebecca Ambrose, Michael R. Beard, Helen Blanchard, Till Böcking, Brendon Chua, Agathe M. G. Colmant, Keaton M. Crosse, Damian F. J. Purcell, Johanna Fraser, Joshua A. Hayward, Stuart T. Hamilton, Matloob Husain, Robin MacDiarmid, Jason M. Mackenzie, Gregory W. Moseley, Thi H. O. Nguyen, Miguel E. Quiñones-Mateu, Karl Robinson, Chaturaka Rodrigo, Julio Rodriguez-Andres, Penny A. Rudd, Anja Werno, Peter White, Paul Young, Peter Speck, Merilyn Hibma, Heidi E. Drummer, Gilda Tachedjian

**Affiliations:** 1 Department of Physiology, Anatomy and Microbiology, School of Life Sciences, La Trobe University, Bundoora, VIC 3086, Australia; k.helbig@latrobe.edu.au (K.J.H.); kcrosse@students.latrobe.edu.au (K.M.C.); 2 Viral Immunology Systems Program, The Kirby Institute, and School of Medical Sciences, University of New South Wales, Sydney, NSW 2052, Australia; r.bull@unsw.edu.au (R.A.B.); c.rodrigo@unsw.edu.au (C.R.); 3 Host Pathogen Interactions, Centre for Innate Immunity and Infectious Diseases, Hudson Institute of Medical Research, Clayton, VIC 3168, Australia; rebecca.ambrose@hudson.org.au; 4 School of Biological Sciences, Faculty of Sciences, University of Adelaide, Adelaide, SA 5005, Australia; michael.beard@adelaide.edu.au; 5 Institute for Glycomics, Griffith University, Southport, QLD 4215, Australia; h.blanchard@griffith.edu.au (H.B.); p.rudd@griffith.edu.au (P.A.R.); 6 ARC Centre of Excellence in Advanced Molecular Imaging & EMBL Australia Node in Single Molecule Sci., University of New South Wales, School of Medical Sciences, Sydney, NSW 2052, Australia; till.boecking@unsw.edu.au; 7 Department of Microbiology and Immunology, The Peter Doherty Institute for Infection and Immunity, University of Melbourne, Melbourne, VIC 3000, Australia; bychua@unimelb.edu.au (B.C.); dfjp@unimelb.edu.au (D.F.J.P.); jason.mackenzie@unimelb.edu.au (J.M.M.); thonguyen@unimelb.edu.au (T.H.O.N.); julio.rodriguez@unimelb.edu.au (J.R.-A.); heidi.drummer@burnet.edu.au (H.E.D.); 8 Australian Infectious Diseases Research Centre, School of Chemistry and Molecular Biosciences, The University of Queensland, St. Lucia, QLD 4072, Australia; agathe.colmant@uq.net.au (A.M.G.C.); p.young@uq.edu.au (P.Y.); 9 World Mosquito Program, Monash University, Clayton, VIC 3800, Australia; johanna.fraser@monash.edu; 10 Burnet Institute, Melbourne, VIC 3004, Australia; joshua.hayward@burnet.edu.au; 11 Department of Microbiology, Monash University, Clayton, VIC 3800, Australia; greg.moseley@monash.edu; 12 Serology and Virology Division, Department of Microbiology, NSW Health Pathology, Prince of Wales Hospital and School of Women’s and Children’s Health, Faculty of Medicine, University of New South Wales, Sydney, NSW 2031, Australia; Stuart.Hamilton@health.nsw.gov.au; 13 Department of Microbiology and Immunology, University of Otago, Dunedin 9054, New Zealand; matloob.husain@otago.ac.nz (M.H.); miguel.quinones-mateu@otago.ac.nz (M.E.Q.-M.); 14 The New Zealand Institute for Plant and Food Research Limited and School of Biological Sciences, The University of Auckland, Auckland 1010, New Zealand; Robin.MacDiarmid@plantandfood.co.nz; 15 Centre for Horticultural Science, Queensland Alliance for Agricultural and Food Innovation, The University of Queensland, St. Lucia, QLD 4072, Australia; k.robinson2@uq.edu.au; 16 Microbiology Department, Virology/Serology Section, Canterbury Health Laboratories, Christchurch 8011, New Zealand; Anja.Werno@cdhb.health.nz; 17 School of Biotechnology and Biomolecular Sciences, Faculty Science, University of New South Wales, Sydney, NSW 2052, Australia; p.white@unsw.edu.au; 18 College of Science and Engineering, Flinders University, Bedford Park, SA 5042, Australia; peter.speck@flinders.edu.au; 19 Department of Pathology, Otago Medical School, University of Otago, Dunedin 9054, New Zealand; Merilyn.hibma@otago.ac.nz

**Keywords:** indigenous virology, animal viruses, virus–host interactions, clinical virology, immunology, innate immunity, bacteriophages, plant viruses, antivirals, vaccines, systems virology, epidemiology

## Abstract

The Australasian Virology Society (AVS) aims to promote, support and advocate for the discipline of virology in the Australasian region. The society was incorporated in 2011 after 10 years operating as the Australian Virology Group (AVG) founded in 2001, coinciding with the inaugural biennial scientific meeting. AVS conferences aim to provide a forum for the dissemination of all aspects of virology, foster collaboration, and encourage participation by students and post-doctoral researchers. The tenth Australasian Virology Society (AVS10) scientific meeting was held on 2–5 December 2019 in Queenstown, New Zealand. This report highlights the latest research presented at the meeting, which included cutting-edge virology presented by our international plenary speakers Ana Fernandez-Sesma and Benjamin tenOever, and keynote Richard Kuhn. AVS10 honoured female pioneers in Australian virology, Lorena Brown and Barbara Coulson. We report outcomes from the AVS10 career development session on “Successfully transitioning from post-doc to lab head”, winners of best presentation awards, and the AVS gender equity policy, initiated in 2013. Plans for the 2021 meeting are underway which will celebrate the 20th anniversary of AVS where it all began, in Fraser Island, Queensland, Australia.

## 1. Introduction

The 10th Australasian Virology Society (AVS10) meeting was held on 2–5 December 2019 in stunning Queenstown, New Zealand. The inaugural AVS meeting, then known as the Australian Virology Group, was held on Fraser Island, Queensland, in 2001. The Australian Virology Group (AVG) was founded by Professor Paul Young, who led the group for 10 years, holding meetings every two years. The AVG became an incorporated Society (AVS) in 2010 and Professor Young was our first president before passing on the baton to Professor Damian Purcell (AVS President 2011–2015), followed by Professor Nigel McMillan (AVS President 2015–2017) and now Professor Gilda Tachedjian (AVS President 2017–current). Eighteen years later, we marked our 10th AVS scientific meeting by returning to Queenstown, the site of the 2013 (AVS7) meeting. The AVS biennial meeting is the premier virology conference in Australasia, providing a forum to disseminate the latest research on the biology of human, animal, plant or prokaryotic viruses in a relaxed yet engaging environment. AVS aims to foster collaboration between different fields of virology, to strengthen multidisciplinary virus-related research, and to encourage the participation of students and post-doctoral researchers. 

We were delighted to welcome our plenary speaker Dr. Ana Fernandez-Sesma, who delivered the Ruth Bishop Oration on dengue viruses and innate immunity, and Professor Benjamin tenOever, the Robert Webster Orator, who presented on the evolution of antiviral defenses. We were thrilled that Professor Richard Kuhn, our third international speaker, presented a keynote on structural studies of flaviviruses. Professor Kuhn was the plenary speaker at our meeting in 2007 (AVG4) and he was keen to return to the meeting. Professor Lorena Brown and Professor Barbara Coulson, who have made outstanding contributions in virology throughout their distinguished careers in Australia, were honoured at AVS10 as lead invited session speakers to present their research on influenza and rotavirus, respectively. AVS10 featured an additional 12 invited speakers from Australasia, 41 speakers selected from proffered abstracts, 16 early career and student rapid-fire three-minute talks, and over 100 poster presentations. 

**Figure 1:**
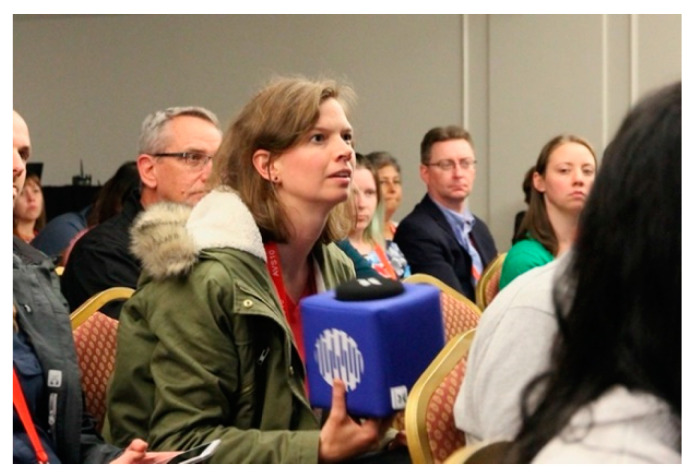
Johanna Fraser asking a question using the “Catchbox” roving microphone at AVS10.

We introduced several new initiatives to promote and support the goals of the society including a session on indigenous research that highlights the disparate burden of virus borne by indigenous communities in Australasia. For the first time at an AVS conference, we used the “Catchbox”, an alternative to a roving microphone that requires a good throwing arm and catching ability of chair and participants, respectively (Figure 1). 

**Figure 2:**
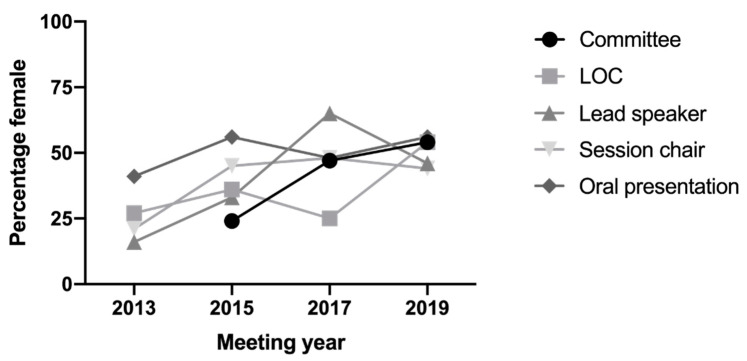
Gender composition within the Australasian Virology Society. Committee, AVS management committee; LOC, local organising committee; lead speaker includes both national and international invited speakers; oral presentation, speakers selected from abstract.

The AVS is committed to gender equity and diversity by aiming to achieve gender balance that reflects our male and female membership in all aspects of the society’s activities including committee membership and scientific meetings. AVS implemented a gender equity policy in 2013, initiated by Professor Gilda Tachedjian and A/Prof Jillian Carr with support from Maree Overall. The AVS policy includes targets, and we monitor our progress against these targets and make them publicly available on our website (www.avs.org.au). We are pleased that the percentage of women on our AVS committee and AVS10 local organising committee (LOC), as well as lead invited speakers, chairs and proffered oral abstracts, has increased to or close to 50%, reflecting the composition of our AVS membership (Figure 2). Our policy was further enhanced in 2019 by Professor Heidi Drummer (AVS Vice President and Gender Equity and Career Development). As a first for an AVS meeting, we offered a parent’s room at AVS10, with live streaming of presentations, to promote the participation of researchers with carer responsibilities, which was used by a number of our delegates. 

**Figure 3:**
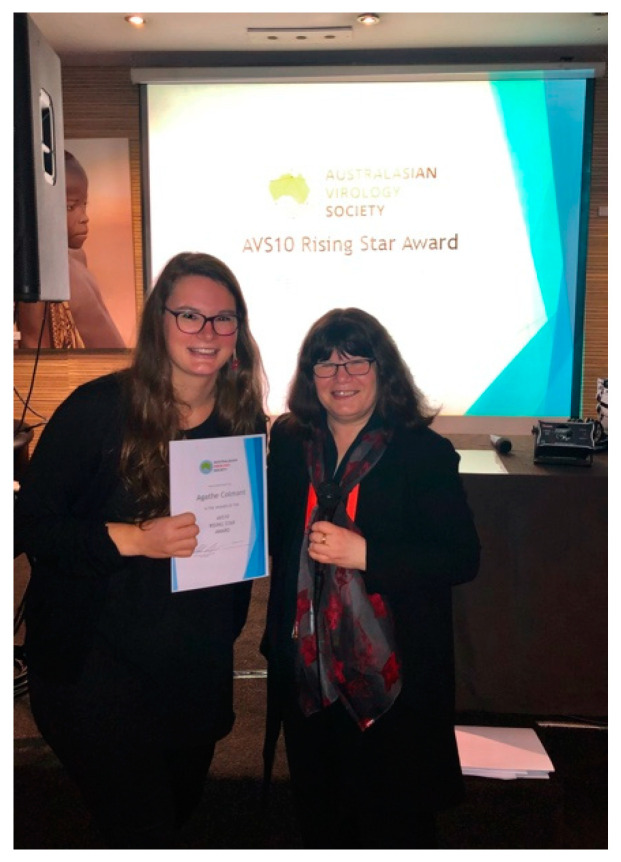
Dr. Agathe Colmant (**left**) receiving the AVS10 Rising Star Award presented by AVS President Professor Gilda Tachedjian (**right**).

Our commitment to supporting the next generation of virology researchers saw the award of 20 prizes and travel awards, made possible through the generous support of our sponsors (Table 1 and Table 2). The winner of our most prestigious AVS prize, the AVS10 Rising Star Award for an early career researcher demonstrating potential as a future leader in the virology discipline, was won by Dr. Agathe Colmant (Figure 3). Dr. Colmant delivered an outstanding presentation on her work on Bamaga virus, a newly discovered novel mosquito-borne flavivirus. 

**Figure 4:**
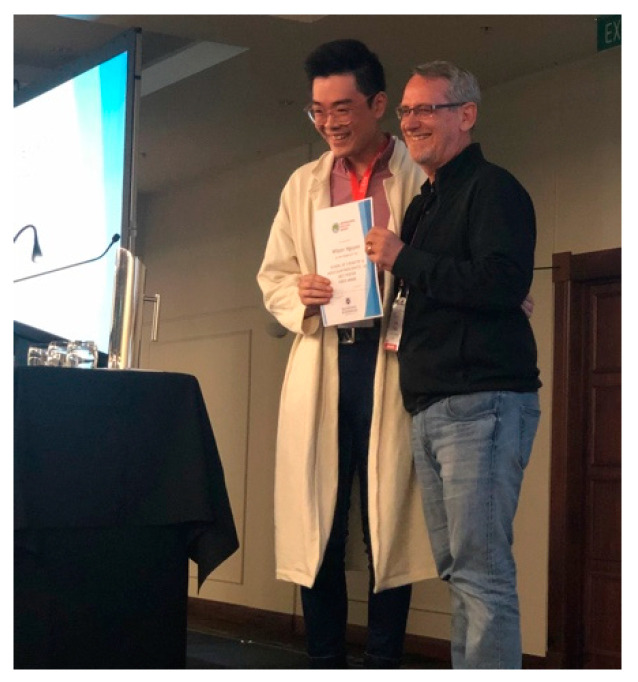
Wilson Nguyen (**left**) receiving the AVS10 Poster Video Award presented by Professor Paul Young (**right**).

Our rapid-fire three-minute presentations provided the opportunity for PhD students and post-doctoral researchers (not more than 5 years) to present on the “big stage” in front of national and international virology experts. AVS10 also introduced the “Poster Video Awards”, where delegates could prepare a three-minute video describing their poster. This educational initiative, championed by Professor Paul Young, aimed to promote skills in science communication, with first prize being awarded to Wilson Nguyen (Figure 4). The videos of the winners were played at the end of the final session of the meeting, which was extremely well received by the audience with the video of the outright winner, Wilson Nguyen, demonstrating remarkable creativity and flair. 

**Figure 5:**
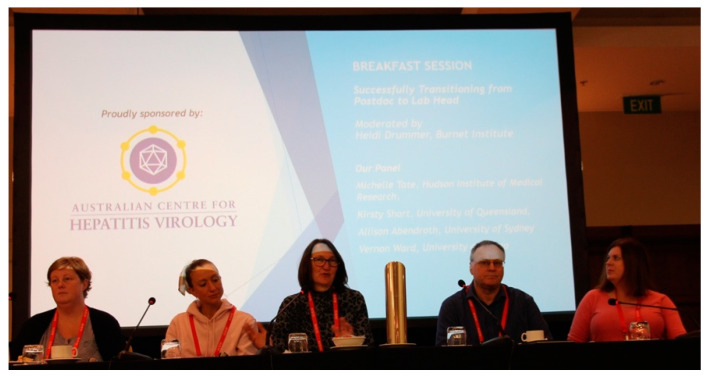
“Successfully transitioning from post-doc to lab head” career development session. Panel members from left to right: Dr. Michelle Tate, Dr. Kirsty Short, Professor Heidi Drummer (moderator), Professor Vernon Ward and Professor Allison Abendroth.

As part of our commitment to career development, we organised a breakfast session on “Successfully transitioning from post-doc to lab head” that included a panel of early, mid and experienced researchers who provided their pearls of wisdom to post-docs, moderated by Professor Heidi Drummer (Figure 5). 

**Figure 6:**
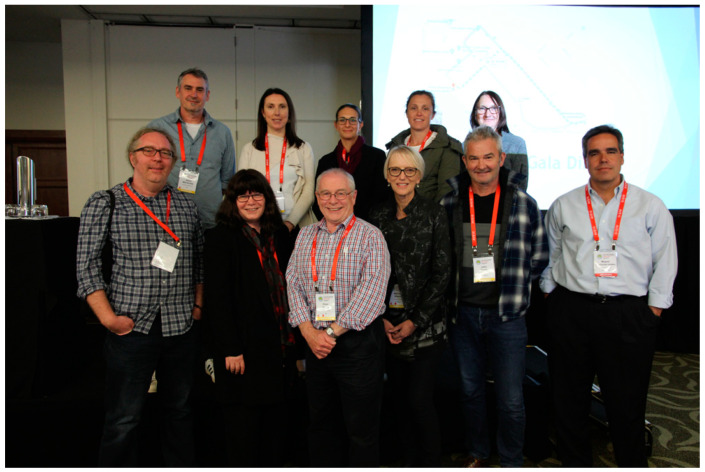
Representative members of the AVS management committee and AVS10 organising committee (top, from left to right): Jason Mackenzie (AVS Treasurer), Karla Helbig, Lara Herrero, Rowena Bull, and Heidi Drummer (AVS Vice President). (Bottom, from left to right): Greg Moseley, Gilda Tachedjian (AVS President), Peter Speck (AVS Secretary), Merilyn Hibma (Convenor), John Taylor (Co-Convenor), and Miguel E. Quiñones-Mateu.

We congratulate and thank the AVS10 2019 organising committee for their efforts in delivering a highly successful meeting led by AVS10 Meeting Convenor, A/Prof Merilyn Hibma and co-Convenor Dr. John Taylor (AVS10 co-Convenor), both based in New Zealand. Members of the organising committee (Figure 6) and their portfolios are listed on Table 3 Our team was ably supported by Michelle Miller and Jody Peters from Leishman Associates, who were instrumental in running a professional meeting. Finally, AVS10 would not have been possible without the generous support from our sponsors. 

## 2. Plenary and Keynote Presentations

### 2.1. Ruth Bishop Oration—Ana Fernandez-Sesma

**Figure 7:**
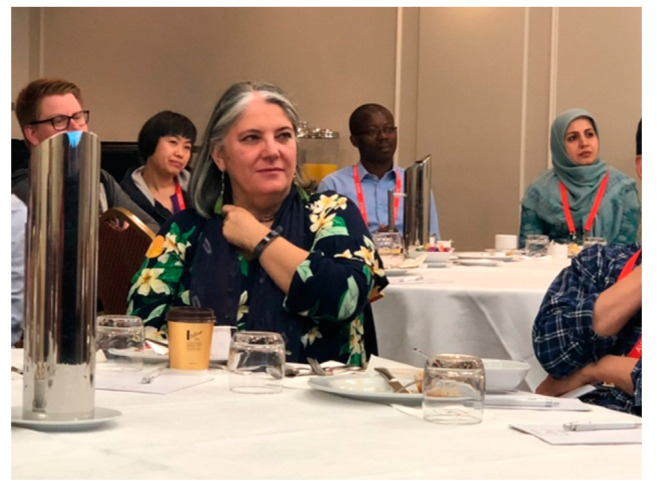
Professor Ana Fernández-Sesma at the AVS10 career development breakfast.

The conference organisers were extremely fortunate in attracting **Ana Fernández-Sesma** (Icahn School of Medicine, Mount Sinai, United States) (Figure 7) to present one of the meeting’s keynote addresses, the Ruth Bishop Oration. Professor Fernández-Sesma and her group are internationally recognised for their seminal work on innate immune responses to flavivirus infections, most notably the countermeasures employed by the dengue viruses (DENV) in escaping this first line of host defense. In this presentation, she highlighted work that identified how immune cells use their DNA-sensing machinery to detect intracellular damage generated early during DENV infection. Two of the crucial innate immune mediators playing a role in this sensing, and that are induced following exposure to mitochondrial DNA (mtDNA), are cyclic GMP–AMP synthase (cGAS) and the stimulator of interferon (IFN) genes (STING). Professor Fernández-Sesma presented evidence that DENV, and specifically the virus encoded protease cofactor NS2B, can efficiently dismantle this sensing mechanism by targeting the cGAS–STING pathway for lysosomal degradation. Her findings show that degradation of these factors, induced during dengue virus infection following leakage of mtDNA, results in the inhibition of type I IFN production and, consequently, antiviral responses. Evidence was also presented showing that other arboviruses may also target the cGAS–STING pathway. Interestingly, different DENV serotypes were shown to induce distinct immune profiles in infected primary cells, with infected and bystander cells both contributing to the overall immune response in DENV infection, by secreting different cytokines and chemokines. Extending these studies into a multidimensional analysis of DENV-2- and DENV-4-infected dendritic cells (DCs) revealed distinct infection kinetics and immune profiles in DCs that may contribute to pathogenesis and transmission of these viruses. It is turning out to be a complicated story, and one that Professor Fernández-Sesma’s team is focused on unravelling.

### 2.2. Robert Webster Oration—Benjamin tenOver

**Figure 8:**
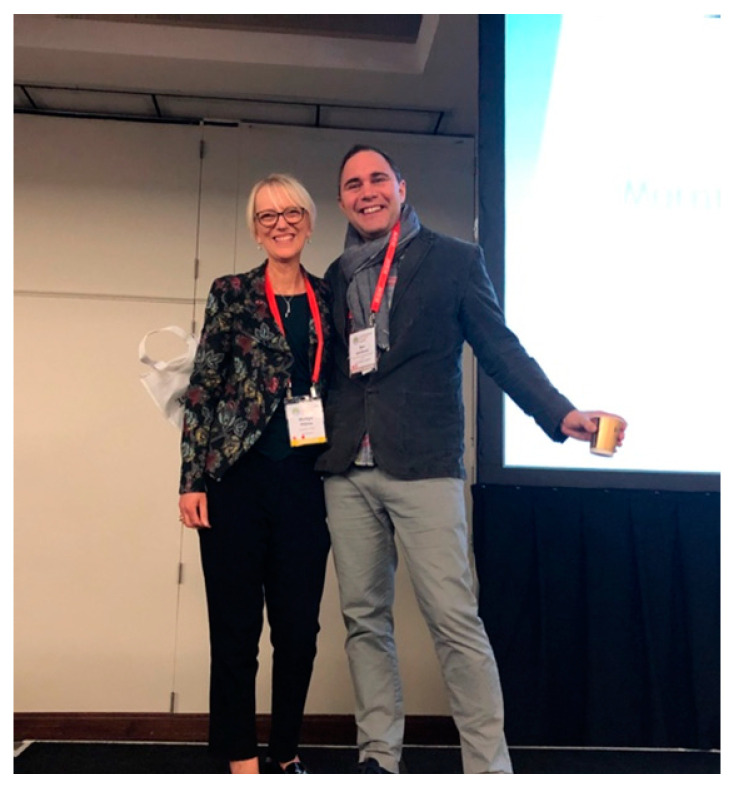
Professor Ben tenOever and A/Prof Merilyn Hibma after the Robert Webster Oration.

A thought-provoking keynote presentation was delivered by invited international speaker, **Benjamin tenOever** (Icahn School of Medicine, Mount Sinai, New York, NY, USA) (Figure 8), who examined the implications of the evolution of RNA-mediated antiviral defenses from lower order prokaryotes, to simple eukaryotes through to higher mammals. During evolution, RNA-guided CRISPR defenses highly effective against DNA viruses, and accounting for 99% of prokaryote viruses, were replaced by RNA interference mechanisms. These mechanisms use short dsRNAs to guide the targeting of RNAse III enzymes to quell the RNA viruses that dominantly infect multicellular eukaryotes, and ultimately in mammals was replaced by interferon-mediated responses to viral DNA or structured RNA. While the ancient RNAi mechanisms were adopted into the miRNA pathway that offers fine control of gene expression, the presentation questioned why human viral defense came to depend largely on an interferon-mediated response to a wide variety of pathogen molecular patterns. Furthermore, did this evolution eliminate the utility of the ancestral RNA-mediated antiviral pathways? In a series of playful experiments that code tandem miRNA targets into mammalian (−)ssRNA *paramyxoviruses* and *orthomyxoviruses*, Ben tenOever showed that the RNA interference pathways used Dicer and Ago2, functioning only for miRNA biogenesis, were able to effect full control of virus replication. For (+)ssRNA viruses, where the target exists in incoming viral genomes, viral replication was slowed, but not eliminated, by the miRNA pathway acting for RNAi. Replication delay resulted from the short lag used precisely to excise the target miRNA sequences from the viral genome, probably due to homologous recombination. Targeting RNAi to nucleoprotein-coding RNA of (−)ssRNA viruses additionally leads to an induction of interferon response, whereas targeting to other regions, such as RNA-dependent polymerase, did not. These experiments of curiosity demonstrate that fully functional RNA defense pathways exist in vertebrates that may be limited by the lack of sufficiently active RNA-dependent RNA polymerases needed to produce the quantity of RNA effectors, especially for larger animals, or a mutually exclusive incompatibility between RNAi and interferon pathways. These findings have implications for new antiviral strategies in humans.

### 2.3. Keynote—Richard Kuhn

**Richard Kuhn** (Purdue University, Indiana, United States) presented a keynote lecture detailing his group’s recent re-examination of flavivirus structures solved by cryo-electron microscopy [1]. Kuhn hypothesised that flaviviruses do not have exact icosahedral symmetry, and that the icosahedral symmetry constraints previously applied to cryo-electron microscopy structures for these viruses may not provide the flexibility required for the large conformational rearrangements that occur during the virus lifecycle. When icosahedral symmetry constraints were excluded in the cryo-EM reconstruction of immature Kunjin virus, they found that the nucleocapsid core touched the inside of the viral lipid membrane at the “proximal pole” and was asymmetrically positioned within the lipid bilayer envelope. The outer glycoprotein spikes on the “distal pole” were either distorted or missing. In the asymmetric reconstruction of mature Kunjin virus, the core was re-positioned, concentric with the glycoprotein shell and there remained a distortion of the glycoproteins on one pole of the virion. This implies that the glycoproteins have a geometric defect that perhaps facilitates the transitions that occur during maturation. Kuhn suggested that this defect in number and arrangement of the glycoproteins may reflect the consequence of membrane budding.

## 3. Viral Epidemiology and Diagnostics

In this session, **Allison Imrie** (University of Western Australia, Perth, Australia) spoke of the emerging threat of dengue virus in Australia driven by increasing international travel to and from endemic countries, mainly Indonesia. The number of infections detected in Western Australia, mainly involving DENV serotypes 1 and 2, has been on the rise over the past decade, except for 2017/2018, when an unexplained decline occurred. The numbers have surged again in 2019. A prospective follow up of infected patients in Western Australia has been established to observe neutralization antibody responses against homologous and heterologous viruses from one-week post-infection to up to seven years. Data shows that although antibody-mediated neutralization persists against the autologous virus, it wanes over time for within-serotype homologous viruses, questioning the dogma of lifelong immunity against the infecting serotype.

**Narayan Gyawali** (QIMR Berghofer Medical Research Institute, Brisbane, Australia) presented work on a novel scaled-down version of the standard plaque reduction neutralization test (PRNT), re-named as micro-PRNT. He demonstrated its potential utility to sample blood from mosquitoes as an indirect measurement of the prevalence of immunity against Ross River virus (RRV) infection in vertebrate hosts. This assay circumvents the need to directly sample from animals to establish changes in Ross River virus reservoirs in the wild.

**Alice Michie** (University of Western Australia, Perth, Australia) presented the most comprehensive phylogenetic analysis of RRV in Australia to date, generating and analyzing 94 new full-genome sequences (and 12 sequences from public databases). The analysis dated the most recent common ancestor of currently circulating variants at approximately 94 years prior and challenged the traditional geographical classification of RRV lineages. The work demonstrated periodic bursts of genetic diversity in RRV phylogenies with four distinct genotypes within Australia, with genotype 4 being the contemporary circulating variant.

**Nasir Riaz** and **Chaturaka Rodrigo** (University of NSW, Sydney, Australia) introduced a novel wet and dry lab (bioinformatics) workflow to optimise nanopore sequencing to identify within-host variants of hepatitis C virus (HCV). They demonstrated that nanopore sequencing is as accurate as state of art Illumina sequencing for viral consensus sequence generation, providing a coverage of at least 300 reads. Furthermore, nanopore sequencing can reduce sequencing costs to $24–$40 AUD per sample if multiple samples are pooled (multiplexing up to 96 samples) in a single run. The novel bioinformatics pipeline was introduced to differentiate within host variants of HCV by phylogenetically arranging closely related subject specific nanopore reads into clusters and generating a mini-consensus per cluster.

**Grace Yan** (University of NSW, Sydney, Australia) presented work on the utility of wastewater analysis of norovirus and adenovirus sequences and compared the findings with those from clinical samples during the same period in 2018/2019. She argued that data from clinical samples may be skewed towards more severe disease as only symptomatic patients seek treatment, while sampling from wastewater allows a better representation of circulating strains. The genotypes and strains identified from clinical samples demonstrated similar patterns but were not identical in abundance to genotypes and strains identified from wastewater samples. Sampling both sources for viral variants can provide better data for understanding the true prevalence of circulating enteric viruses.

## 4. Viral Immunology

The invited speaker for this session was **Barbara Coulson** (University of Melbourne, Australia), who gave an overview of her career in Rotavirus research. This was tightly woven with discovery of rotavirus by Ruth Bishop, Ian Holmes and others, and an outline of her role in the development of an antibody-based diagnostic test for rotavirus. Professor Coulson’s additional advances in rotavirus research included identifying that IgA in stool is protective against reinfection in children, as well as identification of the host receptors for rotavirus entry. The remainder of the talk focused on her research in mice, which has detailed the immunopathological process that leads to rotavirus induced diabetes. Her mouse model has shown that during the initial phase of rotavirus infection, rotavirus induces bystander activation, via TLR7, resulting in strong activation of plasmacytoid dendritic cells and strong upregulation of interferon-dependent gene expression with a skewed Th1-specific response, resulting in activation of pre-existing islet autoreactive lymphocytes.

**T. H. Oanh Nguyen** (University of Melbourne, Australia) presented work detailing the adaptive immune response to the inactivated influenza vaccine (IIV) [2]. Using antigen-specific high-dimensional flow cytometry, she described the longitudinal expansion and contraction of three main B cells subsets: CXCR5-CXCR3+ ASC population, CD21hiCD27+ memory B-cells and CD21loCD27+ B-cells. The emergence of T follicular helper (Tfh) cells was shown to correlate with the emergence of both memory B cell subsets. The group then confirmed that vaccine induced responses were comparable to natural influenza immune responses. The natural immune responses were also found to be enriched for both IgG and IgA B cells during acute infection and by examining where these immune responses were localised suggested that effective vaccines will need to induce Tfh responses and illicit memory B cell responses that reside in the lungs and secondary lymphoid tissues.

**Carolyn Samer** (University of Sydney, NSW, Australia) presented evidence that the herpes simplex virus 1 (HSV-1) rapidly downregulates both cellular and surface MHC I-related gene protein, MR1 [3], the antigen presentation molecule that is recognised by the T cell receptor on MAIT T cells. Whilst several viral gene products are likely to be involved in this immune modulation, the HSV-1 Us3 gene product is implicated in the loss of surface MR1. These findings illustrate for the first time that a pathogen can disrupt antigen presentation by MR1 and subsequent MAIT cell activation and thus expands our knowledge of the immunomodulatory mechanisms adopted by viruses.

**Andreas Suhrbier** (QIMR Berhofer Medical Research Institute, Brisbane, Australia) presented his novel findings surrounding the counter intuitive role of a high-fibre diet in the exacerbation of Chikungunya viral infection [4]. High-fibre diets and their gut fermented by-products are known to reduce inflammation in non-infectious settings; however, Andreas’s team has now demonstrated that high-fibre diets, which results in the production by gut microbiota of the short-chain fatty acid butyrate, significantly enhanced viral driven cytopathic effects. Mice fed high-fibre diets were found to have enhanced edema around peripheral joints, which was underpinned by an upregulation of IL-17 and subsequent neutrophil recruitment.

The final speaker for this session was PhD student, **Alexander Underwood** (UNSW, Australia). He described a human cohort of hepatitis C virus-infected individuals and that antigenic sin, the recall of previous immune responses rather than new immune responses, was commonly observed in reinfection of hepatitis C virus. This may be problematic for hepatitis C virus, which consists of a highly genetically diverse genera. His work has demonstrated that broad neutralizing antibodies on their own were not associated with protection, but instead the quality of the memory B cells and their ability to proliferate, which may be a better marker of protection against reinfection.

## 5. Clinical Virology

This session opened with a keynote lecture from **Ed Gane** (New Zealand Liver Transplant Unit, Auckland Hospital, New Zealand) entitled “Eradication of chronic hepatitis B virus (HBV) infection—translating clinical virology into practice.” Global elimination of HBV has become a reality due to the availability of a safe, effective and inexpensive vaccine but 300 million adults currently live with chronic HBV infection. Although nucleoside polymerase inhibitors can maintain viral suppression and reduce complications, life-long therapy is associated with high cost, risk of breakthrough and potential toxicity. Other steps of the HBV lifecycle can be targeted by small molecules including inhibitors of entry, capsid and virion assembly/release. Capsid assembly modulators (i) prevent encapsidation of pregenomic RNA (pgRNA) and block HBV replication; and (ii) inhibit de-novo formation of covalently closed circular DNA (cccDNA) by interfering with disassembly of the capsid. These inhibitors have recently been demonstrated to be synergistic with nucleos(t)ide analogues (NUCs) in reducing HBV DNA, pgRNA and HBsAg. Translation inhibitors such as small interfering RNAs and antisense oligonucleotides can silence multiple HBV transcripts, potentially blocking the production of the core, polymerase, surface, X-protein. The first clinical studies show potent suppression of HBsAg with only three doses. Chronic HBV infection is characterised by high viral load and antigen burden and inadequate host immune responses. Innovative approaches to restore innate and adaptive immune responses against HBV are currently in clinical development and include therapeutic vaccines, RIG-I agonists, and TLR-7 and TLR-8 agonists. Pilot studies with checkpoint inhibitors, which reverse T cell exhaustion, have also produced promising results. It is likely that HBV Cure will require combinations of novel immunomodulatory, antiviral and cccDNA silencing strategies. Efficacy, safety, route of administration and cost will ultimately determine the impact of these new regimens on the burden of HBV.

**Dongsheng Li** (QIMR Berghofer Medical Research Institute, Brisbane, Australia) discussed research on a novel antiviral to combat dengue virus (DENV) infection, disease and transmission. There is currently no specific DENV antiviral available and the first dengue vaccine licensed has limited data on use and effectiveness. The group’s early research has identified DENV defective interfering particles (DIPs) from all 4 serotype DENV-infected patients. DIPs are virus-like particles that contain the same viral proteins as wild-type virion, and a defective, largely internal deleted viral defective genome (DI RNA). DI RNA can replicate with the help of wild-type virus, while inhibiting wild-type virus replication. DIPs can co-transmit and co-evolve with wild-type virus to produce a durable and resistance-proof antiviral. The group’s early results indicated that DIPs were transmissible and strongly inhibited DENV replication in vitro in serotype-cross and dose-dependent manners, suggesting DENV DIPs may represent a paradigm-shift in strategy of DENV treatment and prevention. The preclinical trials for using DENV DIPs to treat DENV infection and to attenuate DENV transmission in vivo are scheduled.

**Kanta Subbarao** (WHO Collaborating Centre for Reference and Research on Influenza and University of Melbourne, Melbourne, Australia) posed the question: what do post-vaccination antibody kinetics suggest about the timing of the seasonal influenza vaccine? The aim of this study was to estimate the optimal timing of influenza vaccination using antibody decay curves in different age groups, relative to the start, peak, and end of previous influenza epidemics. The final sample consisted of 71 adults (staff aged 18–50 year), 15 ambulatory elderly (hospital volunteers aged 65 year+) and 16 frail elderly (aged care residents aged 65 year+). Estimated geometric mean titres (GMTs) increased for all viruses, peaking 1 month post-vaccination in all groups, before the peak of the season. Titres declined at 3 months, thereafter remaining steady but above baseline, with 6 month post-vaccination GMTs 1.4-, 1.9-, 1.9- and 1.9-fold above baseline for A(H1N1)pdm09, A(H3N2), B/Yamagata and B/Victoria, respectively. The pattern of antibody decay was not significantly different between age groups. The data suggest that subtype-specific antibody-mediated protection following vaccination persists throughout the typical influenza season.

**Kirsty Short** (The School of Chemistry and Molecular Biosciences, University of Queensland, Brisbane, Australia) presented research demonstrating that a history of obesity reduces the immune response to influenza virus in an NLRP3-dependent manner. Obesity significantly increases the risk of death following an influenza virus infection. A novel mouse model was developed to study the long-term effects of obesity on antiviral immunity. Upon infection with influenza virus (A/Auckland/09(H1N1)), previously obese (PO) mice displayed increased viral replication, inflammation, body weight loss and pulmonary dysfunction compared to lean-fed mice. Cells in the lung lumen of PO mice also had an altered metabolic state compared to those of lean fed mice. Importantly, in mice deficient in the NLRP3 inflammasome, obesity had no long-term effect on susceptibility to influenza virus infection. The group propose that obesity can have long-term, NLRP3-dependent, effects on the metabolism of innate inflammatory cells such that they are impaired in their antiviral response.

**Michelle Tate** (Hudson Institute of Medical Research, Clayton, Australia) posed the question: could a World War II drug be repurposed to limit viral hyperinflammation? Severe influenza A virus (IAV) infections are associated with damaging hyperinflammation that can lead to mortality. The group have identified two existing drugs; Probenecid (used to extend the life of penicillin and is currently used to treat Gout) and AZ11645373 (used for treating inflammatory diseases such as arthritis) which target P2X7 receptor signaling and dampen NLRP3 inflammasome responses during severe IAV infection. Intranasal therapeutic treatment of mice displaying severe influenza disease reduced proinflammatory cytokine production, cellular infiltrates in the lung and provided protection against disease. Importantly, Probenecid and AZ11645737 could be administered at either early or late stage of disease and provide therapeutic efficacy. The group propose these drugs are effective as they dampen NLRP3 responses to a level that is not detrimental or damaging, but sufficient to provide protection.

## 6. Indigenous Virology

**Figure 9:**
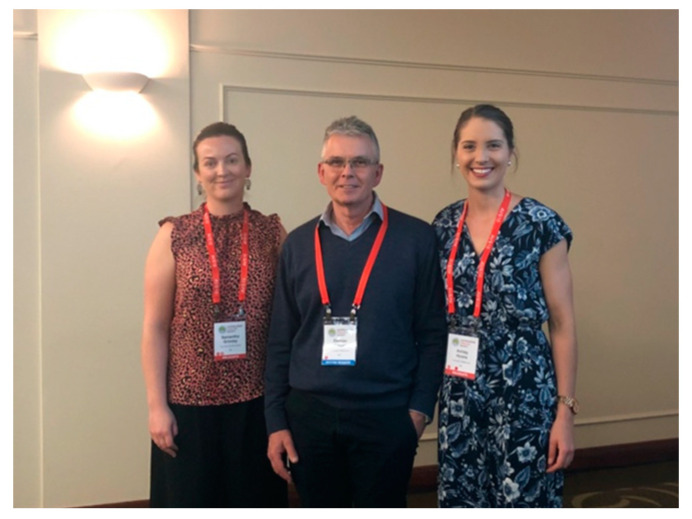
Inaugural Indigenous Virology Session speakers (from left to right), Samantha Grimley, Damian Purcell and Ashley Hirons.

The Inaugural Indigenous Virology Session was held to highlight the important research undertaken by our Indigenous researchers and/or on viruses affecting our Indigenous communities. In this first iteration, the session was spearheaded by **Damian Purcell** and his research team, **Samantha Grimley** and **Ashley Hirons** (University of Melbourne, Australia) (Figure 9), who presented on the significant impact and burden of human T cell lymphotrophic virus type-1 subtype-C (HTLV-1c) strain in Australia. Staggeringly, HTLV-1c is prevalent in ~50% of remote Australian Indigenous communities, particularly in Alice Springs and the Northern Territory, and, as the name suggests, typically infects a range of T cell subsets such as CD4^+^, CD8^+^ and γδT cells. Purcell described a unique cohort of 30 HTLV-1c-infected patients recruited at Alice Springs Hospital where peripheral blood mononuclear cells (PBMCs) and DNA were isolated and the HTLV-1c genomes were sequenced. Newly identified spliced mRNA variants of the viral HBZ regulatory and p16 accessory genes were discovered, which may contribute to disease severity (later presented in detail by his PhD student Ashley Hirons). Furthermore, Purcell described how HTLV-1c proviral load (PVL) was quantified in PBMCs and T cells using a novel digital droplet PCR (ddPCR) assay developed by his research team. High PVL in T cells was associated with chronic inflammatory diseases of the lung and potentially linked to the novel mRNA spliced proteins. Dr. Samantha Grimley’s research focused on the seroprevalence of neutralizing antibodies measured in the plasma of 21 patients from the HTLV-1c cohort. Over 70% of these patients displayed neutralizing antibodies to the HTLV-1 envelope protein, assessed in a reporter infection assay, which correlated with patients of higher PVL and disease outcomes. This study was the first to describe HTLV-1c seroprevalence in a unique cohort of Indigenous Australians and is an important step toward future vaccine development. Lastly, PhD student Ashley Hirons presented on the novel truncated HBZ mRNA transcript that was discovered from the genome sequencing data. She presented that this novel variant may affect the regulation of important down-stream transcriptional processes. Overall these novel findings will provide and enhance the understanding of the pathogenesis of HTLV-1c infection and inform therapeutic and prevention strategies. It was also a terrific example of how Australasian virologists can contribute to the awareness and prevention of significant viral infections in our Indigenous communities.

## 7. Unusual Viruses, Phages and Plant Viruses

Invited speaker, **Peter Waterhouse** (Laureate Fellow and Professor of Molecular Genetics at Queensland University of Technology, Australia) spoke on the interaction between Tomato yellow leaf curl virus (TYLCV) and the model plant *Nicotiana benthamiana* including its wild relatives. The virus TYLCV has a DNA genome and belongs to the genus *Begomovirus* of the family *Geminiviridae*. It has a wide host range with a major economic impact on tomato production. Peter and team have utilised this virus to understand more deeply the differences between the laboratory strain of *N. benthamiana* and its relatives collected recently from its endemic centre of origin, Australia. The team identified RDR3 encoded by the lab strain of the host plant as carrying a premature stop codon that conferred hyper-sensitivity to the TYLCV. By contrast, a Queensland ecotype of the plant that did not bear the mutation was highly resistant. Furthermore, RDR amplifies short interfering RNAs and adds a new player in RNAi mechanism. Using this approach, that maximises the useful differences between the *N. benthamiana* ecotypes, Peter’s team is exploring further mechanistic aspects of RNA interference (RNAi) and its impacts on virus–host plant interactions.

**Sassan Asgari** (School of Biological Sciences, University of Queensland, Brisbane, Australia) described the discovery of a new negev-like virus, *Aedes albopictus* negev-like virus (AaINLV). The negeviruses are insect only viruses, with AaINLV isolated from *Aedes albopictus* Aa23 cell line and in wild-caught mosquitoes. Sequencing, showed the AaINLV viral genome is a positive sense RNA molecule, containing 3 ORF’s, flanked by 5′ and 3′ untranslated regions (UTR’s). A report showing *Wolbachia pipientis* is able to limit arbovirus (e.g., Dengue, Zika) infection in insects presented an opportunity to investigate *Wolbachia* (wAlbB) as an agent to limit or suppress infection of the newly discovered AaINLV. Surprisingly, initial investigation by real-time PCR found AaINLV to be present in high copy numbers in Aa23 cells in the presence of wAlbB, suggesting that no suppression of the virus is occurring. Further investigation added weight to the hypothesis that the longevity of association between AaINLV and wAlbB may be a factor in that both wAlbB and AaINLV have effectively learned to “live” with each other due to a long association, whereas, if introduced to each other in cell culture (i.e., short association), the virus and bacterium have to go through a period of “adjustment”. 

**Callum Le Lay** (University of Sydney, Sydney, Australia) presented his PhD research to date using metagenomics to investigate RNA virus diversity in termites and their bacterial symbionts. From this presentation, it was evident that our current knowledge grossly underestimates the diversity of RNA viruses in the environment, with our understanding limited to only a fraction of medically and agriculturally significant members. Focusing on six Australian termite species, Le Lay discovered 98 novel RNA viruses along with 18 novel single-stranded DNA viruses. Subsequent annotation and phylogenetic tree construction established the majority of the viruses occupied basal positions, a testament to the longevity of the association between the RNA viruses and termites. The substance of this “fishing expedition” showed the power of viral metagenomic analysis with a tantalising glimpse into what will turn out to be just the tip of the RNA “virusburg”.

## 8. Animal and Wildlife Viruses

The session opened with an invited presentation by **Tanja Strive** (CSIRO Health & Biosecurity; Centre for invasive species solutions, Canberra, Australia) on the biological control of rabbits in Australia—“an ongoing co-evolutionary arms race”. Two self-disseminating viral biocontrol agents of European rabbits were discussed; myxoma virus (MYXV), which attenuated shortly after its release; and the calicivirus, rabbit haemorrhagic disease virus (RHDV), which appears to be evolving towards high levels of virulence to facilitate insect transmission from virus-laden carcasses. Dr. Strive highlighted that long-term management of rabbit populations will stem from increased research into rabbit caliciviruses, particularly in the context of recent increases in the genetic diversity caliciviruses in Australia and New Zealand.

The theme of biological control of rabbits was continued with a presentation by **Janine Duckworth** (Manaaki Whenua—Landcare Research, Lincoln, New Zealand), which detailed the effects of RHDV1 K5 release in New Zealand to control wild rabbits. In this study, it was shown that the Czech strain of RHDV1 (released in 1997) was active at all study sites prior to RHDV1 K5 release and that both virus strains were present post-release of RHDV1 K5. The average rate of spread of RHDV1 K5 was 3.5 km/month, with 64%–67% of rabbit carcasses testing positive to RHDV1 K5. This resulted in a 30%–40% reduction in rabbit numbers in the 6–8 weeks following RHDV1 K5 release.

**Joshua A. Hayward** (Burnet Institute, Melbourne, Australia) presented the discovery and characterisation of a group of gammaretroviruses that were found in Australian and Asian bats and are closely related to the koala retrovirus (KoRV) [5]. Using a synthetic proviral molecular construct of one of these bat viruses (Hervey pteropid gammaretrovirus—HPG), it was demonstrated that HPG can infect bat and human cells and displays morphological and enzymatic gammaretrovirus characteristics. These data, combined with serological evidence and the detection of HPG sequences in Australian bats, suggests that bats are reservoirs of KoRV-related retroviruses.

**Karla Helbig** (La Trobe University, Bundoora, Australia) spoke on the differential immune responses of New Zealand paua and Australian hybrid abalone to Haliotid herpesvirus 1 (HaHV-1), which causes abalone viral ganglioneuritis (AVG). The research showed that the HaHV-1-resistant päua, upregulated differentially expressed genes in the haemocytes that are involved in extracellular matrix remodelling upon viral challenge. In contrast, these transcripts were absent in the HaHV-1-susceptible abalone.

**Alice Russo** (University of New South Wales, Sydney, Australia) presented data on comparisons of the viral prevalence and diversity of cane toads (*Rhinella marina*) found in their native range (French Guiana) and two introduced ranges (Australia and Hawaii). In the native range toads, multiple phylogenetically distinct viruses were detected that were not present in the Australian cane toads. These data provide a foundation for studies looking at the influence of viruses on cade toad invasion.

## 9. Virus–Host Interactions I

The first of two sessions on virus–host interactions highlighted strategies evolved by viruses to exploit proteins and small molecules co-opted from the host cell to facilitate steps in the viral lifecycle or molecular defense mechanisms mounted by the host cell to interfere with viral replication. 

Alexander Khromykh (Australian Infectious Diseases Research Centre, University of Queensland, Brisbane, Australia) presented two high-throughput methodologies used in elucidating determinants of flavivirus pathogenesis and evasion of host response. In the first approach, he used deep mutational scanning to identify viral protein function in replication and interactions with host factors [6]. In the second approach, he screened large viral libraries generated by a novel circular polymerase extension reaction method. These methods showed that adaptations on the ZIKV E protein determined adaptations to either mammalian or mosquito cells. In addition, E protein mutants were shown to have temperature-dependent restrictions.

Agathe Colmant (University of Queensland, Brisbane, Australia) presented a new host restriction mechanism of Bamaga virus, a newly discovered flavivirus that replicates in mosquitoes. Interestingly, replication in vertebrate cells was attenuated at 37 °C but restored at 34 °C, whereby the temperature-dependent restriction was observed after cell entry. Adaptive mutations that allowed replication at the higher temperature were mapped to residues close to a viral protease site suggesting that suppression of proteolytic processing prevents replication of Bamaga virus in vertebrate cells.

K M Rifat Faysal (UNSW, Sydney, Australia) presented new single-molecule fluorescence imaging approaches to investigate the uncoating kinetics of HIV-1 capsids. Analysis of capsid mutations in this assay allowed identification of residues involved in selectively binding the host molecule inositol hexakisphosphate (IP6) as assembly factors. IP6 stabilises the immature virus during assembly in the producer cell and is then recycled to catalyse assembly of the mature capsid inside the viral particle, revealing a role of this host molecule at multiple stages of the viral lifecycle.

Jade Forwood (Charles Sturt University, Wagga Wagga, Australia) and his team determined how Hendra and Nipah viruses hijack cellular machinery for import of viral W proteins into the nucleus. Nuclear localisation of W proteins is required to counteract innate immune responses that would otherwise prevent the virus from replicating. X-ray crystal structures of complexes between nuclear import machinery and the nuclear localisation sequence of W proteins revealed the residues involved in the high-affinity interaction with the transport receptor importin α3, adding to our understanding of virus–host interactions and nuclear transport adaptor cargo specificity.

Robin Macdiarmid (Plant and Food Research, Auckland, New Zealand) presented on the reciprocal control of replication between a new ssDNA mycovirus and its host. The team identified and purified infectious viral particles of an exotic virus, named *Botrytis gemydayivirus* 1, which represents a new genus and species. The virus is mechanically transmitted and infects the *Botrytis cinerea*, a fungus that leads to disease in plant species including grapes. The new virus restricted the growth of its fungal host.

## 10. Systems Virology and Viromes

Invited speaker, **Jeremy Barr** (Monash University, Clayton, Australia), emphasised that bacteriophages are one of the dominant groups of biological entities in our body, outnumbering both bacterial and human cells. Despite this observation, very little sequence data is publicly available for bacteriophages and how homologous bacteriophage species are between people. To remedy this knowledge gap, Dr. Barr has employed the bioinformatics tool, VirSorter, to identify new bacteriophages. He has also studied bacteriophage populations (virome) in humans and identified that these populations are very unique between individuals. This is a new and exciting area of research and, given that bacteria are their natural hosts, phages are an integral member of the human microbiome, and so their potential association with disease is of great interest.

**Jessamine Hazlewood** (QIMR Berghofer Medical Research Institute, Brisbane, Australia), reported on the use of the poxvirus-derived vector system, Sementis Copenhagen vector (SCV), to deliver arboviral structural proteins in mice. Their aim was to utilise RNA-Seq to understand successful signatures of protective immunity, and also, especially in the case of the chikungunya virus, to test for signatures of arthritis. Natural chikungunya virus and a previous chikungunya vaccine have been associated with the development of arthritis. Hazlewood’s aim is to understand RNA signatures associated with adverse vs. safe expression profiles and to use that information to help inform vaccine development. The data so far indicates that signatures of Toll-like receptor activity are associated with SCV immunogenicity.

**Andrii Slonchak** (University of Queensland, Brisbane, Australia) presented work on the importance of non-coding subgenomic flaviviral RNA (sfRNA) in successful replication and transmission of Zika virus, and its subsequent impact on disease pathogenesis. Dr. Slonchak made mutant Zika viruses unable to make sfRNA and then discovered that while they could replicate in vitro, their ability to spread to the salivary glands in vivo was attenuated. Further investigation suggested that while sfRNA had no effect on the RNAi pathway, it is important for preventing cell apoptosis by inhibiting Caspase 7 expression, and preventing innate immune functions by inhibiting phosphorylation of STAT1 [7]. Dr. Slonchak also presented data revealing that production of sfRNA was linked with the suppression of genes including FOXG1, indicating that sfRNA is involved with disruption of brain development. Overall these three talks highlighted the significant findings that can be made by examining both the viral and host responses through a systems virology approach aided by high level genomic data.

## 11. Virus–Host Interactions II

Invited speaker **Karyn Johnson** (University of Queensland, Brisbane, Australia) started off the session by summarizing and sharing new data on the role of host micro RNAs (miRNAs), now commonly-known regulators of gene expression, during virus infections. Karyn emphasised that miRNAs could exert both proviral and antiviral functions during virus infection. By using the natural *Drosophila melanogaster*-Drosophila C virus (DCV) model system, she showed that miR-956, through its target Ect4, supports DCV replication [8], whereas the miR-8 through its effector dJun inhibits DCV replication [9]. Building on these findings, her group screened individual miRNA-knockout Drosophila mutant libraries for impact on infection with three different viruses: DCV, Flock House virus and Sindbis virus. She found that at least half of the miRNAs screened had an impact, either proviral or antiviral, on virus infections. Further, some of these miRNAs were common in their impact on the infections of three viruses. Her group is completing the screen and aiming to identify an impact pattern of miRNAs on the infection of diverse insect viruses.

**Alice McSweeney** (University of Otago, New Zealand) presented her work on murine norovirus genome-linked protein VPg, which is encoded by all noroviruses and known to arrest host cell cycle at the G0/G1 interphase to benefit virus replication. Alice shared that the N-terminal region of VPg is essential but not sufficient on its own for cell-cycle arrest. Furthermore, the VPg N-terminal region possesses a functional positively-charged amino acid motif (KGKxKxGRG) and has the ability to non-specifically bind RNA at low affinity.

**Stephen Rawlinson** (Monash University, Clayton, Australia) highlighted that despite carrying out key replication steps in the host cell cytoplasm, many RNA viruses target their proteins to the host nucleolus. He presented that Nipah virus and Hendra virus M proteins localise to a subnucleolar region and interacts with nucleolar protein, Treacle. This interaction results in the inhibition of Treacle-mediated host ribosomal RNA biogenesis and facilitates virus replication [10]. In addition, M protein localises to other subnucleolar regions which are linked to host stress responses and translational regulation.

**Byron Shue** (University of Adelaide, Adelaide, Australia) presented data from a CRISPR/Cas9 genome-wide knockout screen that identified RACK1 as a proviral host factor for multiple flaviviruses including Zika, Dengue and West Nile. RACK1, a scaffold protein in host cell homeostatic processes, interacts with flavivirus NS1 protein and is associated with recruitment of NS1 to viral replication complex in endoplasmic reticulum.

**Thomas Tu** (Westmead Institute for Medical Research, Sydney, Australia) closed the session by reporting that the integration of hepatitis B virus (HBV) DNA into host cell genome, a step associated with liver cancer progression and viral persistence, is not driven by an active mechanism involving viral proteins. He found that the rate of integration is similar in cells infected with wild-type HBV or a replication-deficient HBV mutant or cells transfected with viral DNA. These data suggest that DNA integration occurs through a passive mechanism involving host cell DNA repair machinery.

## 12. Viral Structures, Receptors, Replication

**David Jacques** (University of New South Wales, Sydney, Australia) gave an elegant talk that described the molecular structure of the HIV capsid complex, where each capsid hexamer has a size-selective pore bounded by a ring of six arginine residues, and the role of a highly conserved arginine amino acid residue (R18). The HIV capsid is a fullerene cone comprising ~250 hexamers and exactly 12 pentamers of the capsid protein. R18 is located at the centre of each hexamer and pentamer. Despite exerting a strong electrostatic force at a critical junction of the capsid lattice, this residue is essential for the function of many retroviruses [11]. Using a variety of assays including an in vitro single-molecule fluorescence imaging assay, Jacques et al. demonstrated this residue to be multifunctional, playing roles in forming the conical capsid shape, regulating nucleotide import, controlling capsid stability, and recruiting host proteins. He noted that this one residue serves to highlight that while we conceptually separate these processes in the virus lifecycle, they are in fact intimately related.

**Svenja Fritzlar** (Monash University, Clayton, Australia) investigated the late stages of the human cytomegalovirus (HCMV) replication cycle, which is currently not well characterised. A library of >100 HCMV mutants was screened for viral mutants that showed typical viral assembly complex formation (a cytoplasmic megastructure associated with late stage HCMV infection), but which were unable to complete the viral lifecycle. In this way, she identified a subset of viral mutants and from this characterised a viral protein as crucial for HCMV egress. These proteins represent a set of novel antiviral targets for inhibiting HCMV spread.

**Daniel Watterson** (The University of Queensland, Brisbane, Australia) described a novel chimeric virus platform which can be used to rapidly solve the structures of flaviviruses by cryo-electron microscopy [12]. Using a newly identified insect-specific flavivirus, Binjari virus, he showed that they could substitute the Binjari virus structural genes with prME structural genes from a wide range of human pathogenic flavivirus. These chimeric viruses were found to grow to high titres in insect cells but were not able to replicate in vertebrate cells, making them suitable for growth in low-containment facilities, where cryo-electron microscope facilities are typically located. Using West Nile Virus Kunjin strain/Binjari virus chimera as a model system, he showed that the virus chimera authentically recapitulates the wild-type virion structure at the level of the fold and quaternary arrangement of the structural glycoproteins. The rapid resolution of flavivirus chimeras possible with this system could aid in identifying virulence-associated structural changes, and inform structure-based design of diagnostics, therapeutics and vaccines.

**Thao Huynh** (The University of Melbourne, Melbourne, Australia) described a model for investigating the interactions between hepatitis B virus (HBV) and host proteins. Using a plasmid-free monomeric HBV (“1-mer”) transfection model which expresses cccDNA-like molecules, the viral genomic reservoir involved in HBV persistence, the researchers asked whether expected viral replication intermediates could be produced following the co-transfection of host factors known to modulate HBV infection (DDBI proteins). By co-transfecting these plasmids into hepatoma cells, including 1-mers from different viral genotypes, Huynh confirmed that this system produced the expected HBV replicative intermediates observed using more conventional in vitro models, but with the advantage of being able to detect cccDNA-like molecules usually absent in equivalent systems. Given the role of cccDNA in driving HBV persistence, this model may present opportunities for future development of novel therapeutics to cure chronic HBV infections.

## 13. Innate Immunity

Invited speaker, **Kate Stacey’s** (School of Chemistry and Molecular Bioscience, the University of Queensland, Brisbane, Australia) first piece of data was a very early photo of the “Three Musketeers” of RNA virology in Queensland–Alex Khromykh, Paul Young and Roy Hall–who have all made significant contributions to virology at the national and international level. Following on from this, she discussed a role for dengue virus NS1 in activation and induction of TLR4 responses. TLR4 is the canonical receptor for bacterial LPS; however, recent studies have shown that viral and host factors during influenza, Ebola and respiratory virus infections can activate TLR4. Stacey presented data showing that insect- and mammalian cell-expressed DENV NS1 drives inflammatory cytokine expression, which could be blocked by competitive inhibitors for LPS and sepsis. She linked this to NS1 disruption of the endothelial barrier both in the gut and the liver, and proposed that TLR4 antagonists may have therapeutic potential against Dengue infections.

**Matloob Husain** (University of Otago, New Zealand) presented on the role of host acetylation machinery during influenza A virus (IAV) infection. Host acetylation is regulated by a delicate balance between histone acetyltransferases (HATs) and histone deacetylases (HDACs), and the presentation proposed a proviral role for HATs, and an antiviral role for HDACs during IAV replication. Focusing on HDAC4, he showed that HDAC4 knockdown enhanced IAV replication and reduced the production of interferon-stimulated genes (ISGs) such as Viperin, ISG15 and IFITM3. Furthermore, IAV degraded HDAC4 transcripts by its endonuclease PA-X, as well as targeting HDAC4 protein by cleavage through host caspase 3 [13].

**Larisa Labzin** (Institute of Molecular Bioscience, the University of Queensland, Brisbane, Australia) discussed the detection of viral infection, and how the location of pathogen sensing could determine the strength of the responses. Using an adenovirus model, she showed that infection of macrophages with antibody-coated adenovirus triggers strong inflammasome responses from damaged lysosomes. She also went on to describe that antibody-coated adenoviruses that escape into the macrophage cytosol could initiate a second wave of inflammasome signaling, and that this was mediated by the intracellular Fc receptor TRIM21 and cGAS.

**Ebony Monson** (La Trobe University, Bundoora, Australia) continued with the intracellular response theme, presenting data that supported an emerging role for lipid droplets in early antiviral responses. Lipid droplet size and number increased with virus infection (ZIKV, HSV-1 and influenza), and with treatment of DNA/RNA ligands. This increase in lipid droplet production correlated with decreased ZIKV replication and enhanced expression of interferon. Interestingly, this was dependent on EGFR signaling, as EGFR inhibitors increased ZIKV production and limited lipid droplet and interferon production. **Monson** finished off her talk with some exciting work elucidating the proteome of purified lipid droplets and discussed the potential for localisation of antiviral factors to lipid droplets as an antiviral hub.

The final presentation was by **Greg Moseley** (Monash University, Clayton, Australia) who provided for the first time, structural data from NMR and super-resolution microscopy, on how the rabies P protein interacts with STAT1 to block the antiviral interferon response. NMR analysis of the P protein and STAT1 complex revealed multiple contact points between the proteins with mutational analysis at these sites preventing STAT1 binding and IFN antagonism. Importantly, insertion of these mutations in a pathogenic field stain of rabies revealed significant attenuation of disease, highlighting the importance of this interaction. This work furthers our understanding of host–virus interactions that block the innate response to infection and may pave the way for possible novel vaccine strategies.

## 14. Antivirals and Vaccines

Invited speaker Lorena Brown (The Peter Doherty Institute at the University of Melbourne, Australia) discussed recent results from her laboratory, in collaboration with colleagues at Oxford University, relating to the molecular determinants of gene segment packaging in influenza virus [14]. She described how, using cross-linking of gene segments in vitro together with deep sequencing of digested products, revealed a network of inter-segment interactions. Contrary to existing dogma, these interactions were not restricted to the previously defined “packaging sequences” at the ends of the segments but occurred throughout their entire length. The pattern of interactions differed between viruses of different subtypes and even strains. Importantly, the observed interactions were numerous, with hundreds being detected in the population of virus particles as a whole. However, some interactions were found at very high frequency, indicating that these were present in the majority of virus particles. One such high-frequency interaction occurred between the NA and PB1 genes of certain H3N2 viruses. Professor Brown described how this interaction was so powerful that it led to co-selection of the H3N2 NA and PB1 genes during reassortment with an H1N1 virus, even though the resulting reassortant progeny were significantly less fit than viruses that contained the N2 gene together with the PB1 gene from the H1N1 strain. This work revealed that influenza virus can utilise different interactions to form a complex containing one of each of the eight gene segments prior to budding. This flexibility provides a mechanism that can allow for reassortment between different influenza viruses, with the caveat that the stronger interactions are preferentially maintained and shape the gene constellations of resulting reassortant progeny. 

Mosquito-borne flaviviruses cause a significant global health burden and have potential for pandemic emergence [15]. During her presentation, Dr. Hobson-Peters (University of Queensland, Brisbane, Australia) described a recombinant platform for the rapid manufacture of chimeric virus particles for several major pathogenic flaviviruses, based on an insect-specific flavivirus (Binjari virus, BinJV). BinJV seems to be quite tolerant for exchange of its structural protein genes (prME) with those of pathogenic vertebrate-infecting flaviviruses such as Zika, (ZIKV), Dengue (DENV) and West Nile (WNV) viruses. She described the successful production of BinJV chimeras displaying structural proteins of over 25 pathogenic flaviviruses, which are structurally and antigenically indistinguishable from the wild-type viruses. More importantly, the chimera viruses are completely replication-deficient in vertebrate cells but replicate efficiently in mosquito cells, providing a safe approach for antigen production. These constructions can be used in high-throughput diagnostic assays, e.g., microsphere immunoassays, ELISAs and virus neutralization assays and vaccine applications (see below), representing a simple, non-infectious to vertebrate cells, approach to generate chimeric flavivirus particles. As described above, multiple viruses from the *Flaviviridae* family (e.g., dengue virus, Zika virus, Yellow fever virus, etc.) continue to cause havoc worldwide. With close to 400 million cases of dengue infections per year and no dengue virus (DENV)-specific antiviral drug approved, vaccination is the most promising tool to control this arboviral disease. Although one tetravalent live-attenuated chimeric vaccine (CYD-TDV) is currently commercially available in some dengue endemic countries; poor efficacy in children and in seronegative individuals has been observed [16]. 

Jovin Choo (University of Queensland, Brisbane, Australia) and colleagues described the use of the BinJV platform described by Dr. Hobson-Peters to develop a new chimeric DENV vaccine. They exchanged the structural proteins of BinJV with two proteins from dengue virus 2 (DENV-2, prM and E) to construct the BinJV/DENV2-prME chimera. Using an AG129 infectious DENV mouse model, they demonstrated the protective efficacy of BinJV/DENV2-prME chimera by showing (i) high production of neutralizing antibodies and (ii) 80% to 100% survival after challenge with lethal doses of DENV-2. This study confirmed the potential use of BinJV chimera constructions to develop novel vaccines—in this case, against dengue virus.

Anjali Gowripalan (The Australian National University, Canberra, Australia) described how the CRISPR/Cas9 system can be effectively harnessed and adapted into a powerful selection tool for recombinant poxviruses. In recent years, CRISPR/Cas9 has enabled the manufacture of recombinant large dsDNA viruses from various families enabling and accelerating mechanistic studies into viral pathogenesis, host–virus interactions and development of novel interventions. For these viruses, CRISPR/Cas9 is used to facilitate homologous recombination, in much the same way as has been widely adopted for engineering genomes in cells and animals. Dr. Gowripalan’s work, however, highlights the unreliability and poor efficiency of this approach for a vaccinia virus, which has a cytoplasmic site of replication. Moreover, she dissected the reasons why this approach does not simply translate to poxviruses. Using this knowledge, a highly efficient and innovative two-stage method for virus generation was developed and tested. This approach was able to reduce the time for virus generation from months to 1–2 weeks, which extends the utility of vaccinia virus as a vaccine vector to situations requiring a rapid response, such as emerging infections and personalized cancer immunotherapeutic strategies.

With close to 37 million people living with HIV-1 worldwide [17], HIV/AIDS continues to be one of the most important pandemics of the last 50 years. The widespread global access to antiretroviral drugs has led to considerable reductions in morbidity and mortality [18,19]; however, no vaccine is currently available despite a myriad of studies and clever designs [20]. Although it is clear that broad neutralizing antibodies (bNAbs) are needed to control HIV-1 replication, induction of bNAbs by HIV envelope glycoprotein-based vaccines has been difficult due to the high genetic variability of the virus. Here, Andy Poumbourios (Burnet Institute, Melbourne, Australia) described a protein engineering approach aimed at fixing soluble HIV-1 glycoprotein trimers in conformations corresponding to those sampled on virions. Mutations known to stabilise the trimer were combined with a glycosylation site mutation that exposes epitopes of bNAbs. One such mutant with intermediate thermostability and epitope exposure elicited broad neutralization activity in guinea pigs, suggesting that the approach is applicable to vaccine design.

## 15. Three Minute Oral Presentations

AVS10 provided the opportunity for numerous early career researcher—many of whom were current PhD students—to present their novel research findings during two sessions of three-minute oral presentations. These sessions encompassed a broad range of virological research, ranging from investigations into livestock viral pathogens, antiviral and vaccine trials, immune surveillance, to basic viral and host research. **Natalia Salazar-Quiroz** (The Peter Doherty Institute for Infection and Immunity at the University of Melbourne, Australia) was the recipient of the Australian Society for Microbiology award for her three-minute oral presentation demonstrating the dual activity of bovine antibodies to broadly neutralize as well as elicit Fc-effector function to HIV-1 envelope glycoproteins, while **Natalee Newton** (Australian Infectious Diseases Research Centre, The University of Queensland, Brisbane, Australia) received the Microorganisms MDPI award for her presentation of the first cryo-EM structure of the insect-specific flavivirus BinJV. Other presentations of note were those from **Tasmin Gordon** (Burnet Institute) and **Fernando Villalon Letelier** (The Peter Doherty Institute for Infection and Immunity at the University of Melbourne), who both presented novel work describing the activity of host antiviral proteins. Tamsin Gordin characterised the unique ability of Egyptian fruit bat tetherin to control both EBOV and MARV viral particle release, while Villalon Letelier described the ability of MARCH 8 to reduce the infectivity of IAV particles by reducing the incorporation of integral proteins into the virion. Overall, these sessions showcased the remarkable research conducted by early career researchers throughout Australia and New Zealand which helps further understand both animal and human viral diseases and their treatment.

## 16. AVS10 Career Development Session: “Successfully Transitioning from Post-Doc to Lab Head”

AVS held its second career development workshop over breakfast with a panel discussion led by AVS Vice President Professor Heidi Drummer (Burnet Institute) with panelists Professor Allison Abendroth (University of Sydney), Professor Vernon Ward (University of Otago), Dr. Michelle Tate (Hudson Institute) and Dr. Kirsty Short (University of Queensland). The experiences of the five panelists were diverse, and this was equally reflected in their diverse views on this important topic.

Every panel member made a conscious decision to become a lab head and pursued funding opportunities relentlessly, showing a high degree of resilience to knock backs and obstacles. The time required post-PhD varied considerably, with some undertaking several post-doc stints, and others stepping straight out of their first post-doc into a lab head position with funded grants. Overseas post-doc experience was not essential amongst this panel, with several never having worked overseas. A university academic appointment can assist significantly, providing essential personal salary support, access to small grants and high-quality students. These opportunities are limited and highly competitive but should be considered even if relocation is required.

Mentoring is often considered an essential component of training and professional development. On this panel, one member believed strongly that their mentor was essential to their development and future independence. However, this was not identified as being essential for the majority of the panelists. Instead, panelists identified a strong advocate as being perhaps more important; someone who actively pushes you onto review panels, into speaking positions, chairing sessions, and positions of influence. Advocates are often already successful, have nothing to lose by passing on an opportunity to you, and they believe in you and the calibre of your work.

All panel members agreed that once funding has been secured, it is tricky to know how much time should be devoted to writing new grants, as this time commitment detracts from writing manuscripts and performing experiments. There was division amongst the panel, with some believing that every funding opportunity should be pursued, and others believing that a period of consolidation is necessary to ensure that funded work is initiated and providing an important respite from the application process. Small grants should not be overlooked, particularly early in your career, as they provide essential seed funds to generate preliminary data for bigger grants. Sometimes two or three small grants can equal the budget of an Australian Research Council (ARC) or Australia National Health and Medical Research Council (NHMRC) grant for one year, providing consumable funds to support students or short-term employees. Look at University and Institute web pages for lists of funding opportunities, track down people previously successful and ask for advice.

An important consideration for all post-docs is the issue of family responsibilities, whether this is having children, looking after children or elderly parents. The importance of flexible work hours was identified by all panel members and participants as essential requirements to maintain a healthy and productive work–life balance. Time management and being efficient assist in making the most of the working day and many identified the importance of a good network of people to provide support as essential success factors.

Overall, every individual must carve their own path to success; there is no golden ticket, there are no set rules and the path is unlikely to be linear. However, resilience was identified as the one key attribute that you must possess and actively display if you are to make the transition from post-doc to lab head. None of the panel members regretted their decision and medical research remains one of the most exciting and fulfilling careers available to those who are scientifically minded.

## 17. Conclusions

Overall, the presentations at AVS10 showcase the breadth and quality of virology being undertaken in the Australasian region, spanning fundamental research, and translational, clinical and epidemiological studies. AVS10 consistently showcases the talent of our budding virologists. Our next meeting in December 2021 marks an auspicious occasion celebrating 20 years of AVG/AVS, and we are planning to hold the meeting in Fraser Island, Queensland, Australia, the location of the very first Australian virology meeting.

## Figures and Tables

**Table 1:** AVS10 Prizes.

	Winner	Presentation Title
**Emerging Leadership Awards:**		
AVS10 Rising Star (Australasian Virology Society)	Agathe Colmant ^1^	The attenuation of flavivirus Bamaga virus in vertebrates is temperature-dependent and linked to viral protease cleavage efficiency
AVS10 Student Award (Australasian Virology Society)	Ebony Monson ^2^	Early Intracellular Lipid Droplet Accumulation Following Viral Infection Is Required for an Efficient Anti-Viral Response
**Research Presentations:**
Monash Biomedicine Discovery Institute Poster Prize	Joshua Deerain ^3^	Programmed Cell Death During Norovirus Infection
	Paulina Koszalka ^4,5,6^	Use of a hollow fibre infection model to study the selection of resistance to the new influenza antiviral drug baloxavir.
Microorganisms Best Oral Presentation Award (MDPI)	Natalee Newton ^7^	The first cryo-EM structure of an insect-specific flavivirus reveals an infectious immature virion and new mechanism for IgM-based flavivirus immunity
Peter Doherty Institute for Infection & Immunity Award for Indigenous Health Research	Ashley Hirons ^3,8^	Novel hbz mRNA of HTLV-1c results in loss of activation domain
Top Early Career Cellular Microbiologist Award (John Wiley & Sons Ltd.)	Anjali Gowripalan ^9^	Subverting the dogma of CRISPR/Cas9 to powerfully select recombinant poxviruses
Maurice Wilkins Centre Student/ECR Presentation Award	Svenja Fritzlar ^5^	Genome-wide molecular screen reveals novel HCMV genes essential for viral egress
AIDRC Student Presentation Award (Australian Infectious Diseases Research Centre)	Byron Shue ^10^	Screening for a “CRISPR” perspective of RACK1 as a critical pan-flavivirus host factor for virus infection
Institute for Glycomics Student Prize (Griffith University–Institute for Glycomics)	Alice Russo ^11^	Viral prevalence and diversity among cane toads (Rhinella marina) in their native and introduced ranges: can viruses impact invasion success?
Department of Microbiology & Immunology, University of Melbourne Presentation Award	Alexander Underwood ^12^	Defining correlates of antibody-mediated protection against HCV reinfection
La Trobe Centre for Livestock Interactions with Pathogens Travel Award	Gervais Habarugira ^13^	Pathogenesis of WNVKUN infection in experimentally infected Crocodylus porosus
Australian Society for Microbiology Award	Natalia Salazar-Quiroz ^3^	Structural and clade differences in HIV-1 Env trimer vaccines affect antibody functionality
School of Chemistry & Molecular Biosciences, University of Queensland, Best Poster Video Awards	Wilson Nguyen ^14^	Establishing, characterising and utilising new adult mouse models of arthritogenic alphaviruses for pre-clinical evaluations of new interventions.
	David Delgado Diaz ^15,6^	Lactic acid produced by an optimal vaginal microbiota promotes cervicovaginal epithelial barrier integrity: implications for HIV transmission
	Hafsa Rana ^16,17^	Developing methods to investigate viral entry into human foreskin

^1^ University of Queensland, School of Chemistry and Molecular Biosciences, Brisbane, Australia; ^2^ La Trobe University, Bundoora, Australia; ^3^ The Peter Doherty Institute for Infection and Immunity, Melbourne, Australia; ^4^ WHO Collaborating Centre for Reference and Research on Influenza, Peter Doherty Institute for Infection and Immunity, Melbourne, Australia; ^5^ Biomedicine Discovery Institute, Monash University, Clayton, Australia; ^6^ Department of Microbiology, Monash University, Australia. ^7^ Australian Infectious Diseases Research Centre, School of Chemistry and Molecular Biosciences, The University of Queensland, St. Lucia, Australia; ^8^ University of Melbourne, Melbourne, Australia; ^9^ John Curtin School of Medical Research, The Australian National University, Canberra, Australia; ^10^ Research Centre for Infectious Diseases, The University of Adelaide, Adelaide, Australia; ^11^ School of Biotechnology and Biomolecular Sciences, University of New South Wales, Sydney, Australia; ^12^ School of Medical Sciences, University of New South Wales, Randwick, Australia; ^13^ School of Veterinary Science, University of Queensland, Gatton, Australia; ^14^ Inflammation Biology Group, QIMR Berghofer Medical, Research Institute, Herston, Australia; ^15^ Disease Elimination Program and Life Sciences Discipline, Burnet Institute, Melbourne, Australia; ^16^ Westmead Institute for Medical Research, Sydney, Australia; ^17^ University of Sydney, Sydney, Australia.

**Table 2:** AVS10 travel awards.

	Winner	Presentation Title
Microorganisms Travel Award (MDPI)	Anjali Gowripalan ^1^	Subverting the dogma of CRISPR/Cas9 to powerfully select recombinant poxviruses
Viruses Travel Award (MDPI)	Joshua Hayward ^2^	Infectious KoRV-related retroviruses circulating in Australian bats
Thermo Fisher Scientific Travel Awards	Alice Michie ^3^	Genome-scale phylogeny and evolutionary analysis of Ross River virus
	Alice Russo ^4^	Viral prevalence and diversity among cane toads (*Rhinella marina*) in their native and introduced ranges: can viruses impact invasion success?

^1^ John Curtin School of Medical Research, The Australian National University, Canberra, Australia; ^2^ Burnet Institute, Melbourne, Australia; ^3^ University of Western Australia, Perth, Australia; ^4^ School of Biotechnology and Biomolecular Sciences, University of New South Wales, Sydney, Australia.

**Table 3:** AVS10 Organising Committee.

Committee Role	AVS10 Committee Role
Merilyn Hibma	Meeting Convener/Invited Speakers Liaison
John Taylor	Meeting Co-convener/Sponsorship
Gilda Tachedjian	Ex officio (AVS President)
Jason Mackenzie	Financial
Peter Speck	Ex officio (AVS9 Meeting Convenor)
Heidi Drummer	Gender Equity and Career Development
Rowena Bull	Abstracts
Karla Helbig	Program/Presentations
Greg Moseley	AVS Awards and Prizes
Lara Herrero	Social Program
Matloob Husain	Posters
Anja Werno	AVS10 Committee Member
